# A Rare Case of a Child with Bended Scarf-Pin in Left Bronchus: Case Report with Systematic Review And Meta-Analysis

**DOI:** 10.31661/gmj.v13i.3597

**Published:** 2024-10-21

**Authors:** Beril Kayrancioglu, Mustafa Azizoglu, Fatma Sarac

**Affiliations:** ^1^ Department of Pediatric Surgery, Başakşehir Çam and Sakura City Hospital, Istanbul, Turkey; ^2^ Department of Pediatric Surgery, Istanbul Esenyurt Hospital, Istanbul, Turkey; ^3^ Istinye University, Department of Stem Cell and Tissue Engineering, Istanbul, Turkey; ^4^ Pediatric Surgery Meta Analysis Study Group , Meta Academy, Istanbul, Turkey

**Keywords:** Hijab Pin, Foreign Body Aspiration, Pin Aspiration, Children, Infant

## Abstract

Background: In this report, we present the case of a 19-month-old female diagnosed with hijab pin aspiration after a week of persistent coughing, along with a meta analysis and systematic review of the relevant literature. Materials and Methods: A comprehensive search was conducted across multiple databases, including Ovid Medline, Cochrane, PubMed, Web of Science, and SCOPUS, yielding 182 records until August 2024. A total of 7 published study and our case included to final analysis. The complication, morbidity, and mortality analysis has been performed using Jamovi software v2.4 MAJOR proportion analysis section. Case Description: A 19-month-old female was admitted to another hospital with a persistent cough complaint. In X-ray left hijab pin has been detected. The patient underwent a succesfull removal with rigid broncoscopy (RB). A total of 71 patients included this meta analysis. The thoracotomy rate was 8%. The bleeding rate was reported as 1.4%. The reoperation rate was reported as 9.8%. The postoperative intubation rate was reported as 1.4%. The calculated complication rate was found to be 5.6% based on the existing literature. The postoperative hemoptysis incidence was calculated as 0%. Mortality was not reported across any included studies. However, the mortality incidence was calculated as 0% based on included studies. Conclusion: Effective and timely intervention is crucial for managing pediatric hijab pin aspiration. Multidisciplinary approaches ensure successful outcomes and prevent serious complications.

## Introduction

Foreign body aspiration (FBA) is a and a major cause of death and illness in children, especially those under the age of two [1][2][3]. From 2001 to 2016, the United States reported 305,814 nonfatal injuries associated with choking in children aged 0 to 19 years [4][5][6]. FBA is an important clinical problem, especially in pediatric populations where the aspiration of sharp objects like headscarf pins presents numerous challenges [7][8]. Wearing headscarves is very common among young women, and the risks associated with aspiration have been well documented in populations with an Islamic tradition [9][10].

Aspiration of foreign objects often occurs when an individual suddenly laughs, talks, or experiences a lapse in concentration [1][2][3][4]. Foreign body aspiration refers to the unintentional inhalation of an object into the airway, which, if not treated promptly, can lead to life-threatening complications. Symptoms vary based on the location and position of the object in the airway. Common signs include persistent coughing, blood in sputum, or breathing difficulties, though some patients may remain asymptomatic. Sharp foreign objects pose a higher risk, as they can puncture the airway. Imaging, such as chest X-ray or CT scan, helps locate and assess the object, with rigid bronchoscopy being the preferred method for removal [9][10].

In this report, we present the case of a 19-month-old female diagnosed with hijab pin aspiration after a week of persistent coughing, along with a meta analysis and systematic review of the relevant literature.

## Case Description

**Figure-1:**
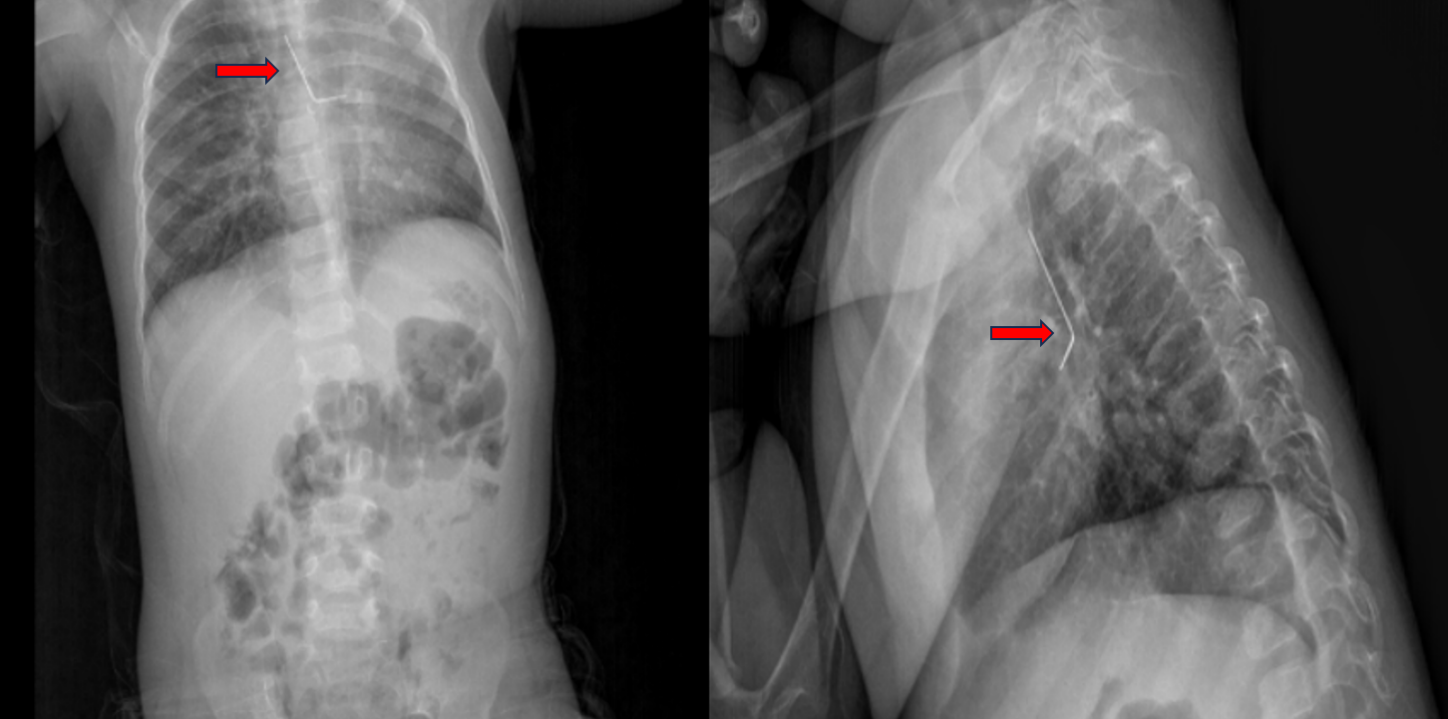
Figure 1. Chest X-ray (the red arrow points to the L-shaped pin)

**Figure-2:**
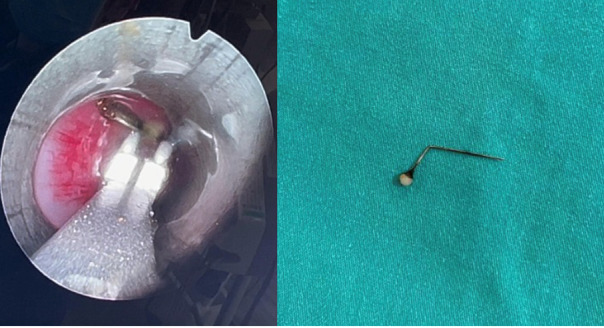
Figure 2. Intraoperative view during bronchoscopy and curved pin after removal

A 19-month-old female was admitted to another hospital with a persistent cough complaint. The foreign body was identified on a chest X-ray. Due to the absence of a pediatric surgeon, the patient was referred to our clinic and subsequently admitted to our care. History of the patient known to have a suspected grape allergy. Her vitals were hemodynamically stable. Oxygen saturation was between 97%-100% at room air. A physical examination revealed diminished breath sounds accompanied by mild wheezing. Laboratory findings were unremarkable. The initial chest X-ray revealed the presence of a foreign object, shaped like an ‘L’, lodged in the left bronchial tree (Figure-1).

The patient underwent a bronchoscopy. From the larynx, we reached the trachea with the bronchoscopy tube. The foreign body was seen on its way to the carina. It was L-shaped, extending from the carina to the left main bronchus. Forceps could not be opened inside the optic sheath because the foreign object was long. Therefore, the camera and forceps were passed through the larynx again without a sheath, and were able to reach the carina. The foreign object was successfully removed using forceps. When we took a second look all paths were clear and the air passage was open. The procedure was successfully performed without any complications or bleeding (Figure-2). The feeding started at the postoperative 6th hour, and the patient was discharged 24 hours after the procedure. The follow-up was uneventful.

## Literature Review

**Figure-3:**
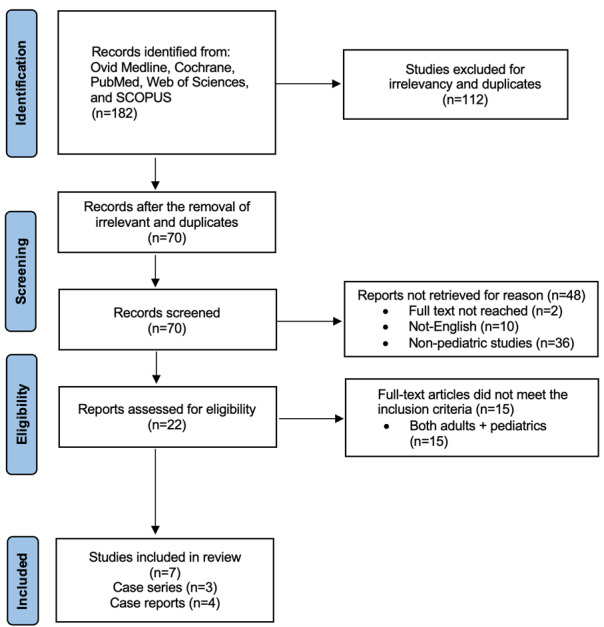
Figure 3. PRISMA flow diagram of study selection

**Table:** Table1. Published studies

**Author**	**Country**	**Study year**	**Publication**	**Type of the study**	**Number of patient**	**Age**
Sheikh *et al*	USA	2020	JAAPA	Case report	1	18 years
Parvez *et al*	UAE	2016	Lung India	Case report	1	11 years
Sarmah *et al*	India	2023	Indian J Head&Neck	Case series	4	13,12,12,13
Murthy *et al*	Oman	2001	Am J Otolaryngology	Case series	6	5,10,16,12,16,16
Harischandra *et al*	S. Lanka	2009	J Laryngolo&Otology	Case report	1	12 years
Maddali *et al*	Oman	2006	Pediatric anesthesia	Case report	1	10 years
Hamouri *et al*	Jordan	2018	J laparoendoscopic Adv	Case series	56	mean: 13.3 years
Our case	Turkey	2024	None	Case report	1	19 months

**Table:** Table2. Summary of included studies

**Author**	**Location**	**Removal way**	**Bleeding**	**Hemoptysis**	**Postop intubation**	**Reoperation**	**Other complications**	**Mortality**
Sheikh *et al*	R	RB	Yes	No	No	No	No	No
Parvez *et al*	L	FB	No	No	No	No	No	No
Sarmah *et al*	L, L, T, NF	RB, TR, TR, RB	Yes (in 1)	No	No	Yes (in 1)	Needed chest drain for 1 week in 1 patient	No
Murthy *et al*	T (1) R (2) L (2) Swallow (1)	RB in all patients	No	No	No	No	No	No
Harischandra *et al*	L	TR	No	No	No	Yes	No	No
Maddali *et al*	R	RB	No	No	Yes	Yes	Contralateral atelectasis	No
Hamouri *et al*	T (7), R (25) L (24)	FB in 52, FB+fluoroscopy in 2, and mini TR in 2	No	No	No	Yes (in 4)	Asthma (in 2)	No
Our case	L	RB	No	No	No	No	No	No

**RB:** Rigid bronchoscope, **FB:** Flexible bronchoscope, **TR:** Thoracotomy, **T:** Trachea, **L:** Left bronchus, **R:** Right bronchus.

Data Extraction

Two researchers (MA and BZK) independently evaluated the studies included in this meta-analysis. Key information such as participant numbers, study design, and publication year was systematically collected. Additionally, data on population characteristics, including patient age at the time of surgery and total number of patients, were extracted. The meta-analysis focused on identifying essential variables, including surgical outcomes, morbidity, mortality, and complications.

Published Studies

The reviewed studies on pediatric hijab pin aspiration of foreign objects include a range of case reports and case series from various countries [11][12][13][14][15][16][17]. A total of 70 cases were found during literature search. Sheikh et al. (2020) and Parvez et al. (2016) each reported a single case involving 18- and 11-year-olds, respectively. Sarmah et al. (2023) presented a series of four cases involving children aged 12 to 13 years. Murthy et al. (2001) described six cases with ages ranging from 5 to 16 years. Other notable reports include Harischandra et al. (2009) and Maddali et al. (2006). The published papers summary were given in Table-1.

In 44% of patients, pin was found in the left bronchus and 41% in right bronchus. The findings of all included studies were given in Table-2.

Sheikh and colleagues published a case report detailing their experience with a single patient. The case involved an 18-year-old female who presented to the Emergency Department 1-2 hours after aspirating a pin. A chest X-ray revealed the pin localized in the right main bronchus. The patient underwent bronchoscopy, which confirmed the presence of the pin in the right main bronchus. The pin appeared to have punctured the superficial mucosa along the medial wall of the bronchus, causing minimal bleeding that resolved spontaneously. The patient was monitored postoperatively without complications and was discharged without any issues. The postoperative follow-up was uneventful [11].

Parvez and colleagues reported a case involving an 11-year-old female patient who was found to have a pin that had pierced the lung parenchyma in the left bronchus. The patient initially underwent rigid bronchoscopy, but the extraction attempt was unsuccessful. Subsequently, the pin was successfully removed using flexible bronchoscopy. The postoperative follow-up was uneventful, with no hemoptysis, bleeding, morbidity, or mortality observed [12].

Sarmah et al. reported 4 cases. Case 1 involved a 13-year-old female who presented with cough and chest pain three hours after accidentally aspirating a headscarf pin while putting on her hijab. The pin was successfully removed from the left main bronchus using rigid bronchoscopy, and there were no postoperative complications. In Case 2, a 12-year-old female presented with a 15-day history of cough and reduced air entry in the left lung following the aspiration of a headscarf pin. After three unsuccessful rigid bronchoscopy attempts, the pin was removed via thoracotomy. The postoperative period was uneventful, with no complications. Case 3 featured a 13-year-old female who experienced severe coughing for four days after aspirating a pin. The initial bronchoscopy failed to remove the pin, but it was successfully extracted from the trachea following a thoracotomy and subsequent bronchoscopy. The patient had no complications during the postoperative follow-up. Case 4 involved a 12-year-old female who presented three days after pin aspiration with a cough and reduced air entry in the right lung. Bronchoscopy did not locate the pin, and it was presumed that the pin had been expelled spontaneously through coughing. There were no postoperative complications observed [13].

Murthy et al. reviewed six cases of sharp foreign body aspiration involving female patients aged 5 to 16 years. These patients were admitted to the ENT wards between 3 hours and 1 day after the accidental aspiration of scarf pins. Rigid bronchoscopy was performed in all cases to remove the pins. The scarf pins were located in the right bronchus in two patients, the left bronchus in two patients, and the trachea in one patient. One patient coughed out the pin, which was later swallowed. The postoperative period was uneventful for all patients [14].

Harischandra et al. published a 12-year-old girl accidentally inhaled a lapel pin after laughing, leading to a brief episode of minimal hemoptysis. A chest radiograph later revealed the pin lodged in the left lung. Despite multiple attempts at removal using rigid and flexible bronchoscopy, the pin could not be retrieved. Ultimately, a posterolateral thoracotomy was performed, successfully locating and removing the pin from the left lung. The patient recovered uneventfully post-operatively [15].

Maddali et al. published a case of a 10-year-old girl who experienced total contralateral atelectasis following prolonged rigid bronchoscopy to retrieve a scarf pin aspirated into her right lung. Despite successful removal of the pin after five hours, the patient developed a complete collapse of the left lung. Factors contributing to this complication included prolonged procedure time, a high BMI requiring greater tidal volumes, significant air leakage during bronchoscopy, and copious lung secretions. The case highlights the need for vigilant monitoring of the contralateral lung during such procedures, emphasizing the importance of proper ventilation, clearance of secretions. The child was successfully extubated after 12 hours of mechanical ventilation, following effective airway management with bronchodilators (salbutamol) and chest physiotherapy. She was discharged home 72 hours later [16].

Hamouri et al. reported a series of 56 cases involving needle aspiration. In 52 cases, the needles were successfully removed using flexible bronchoscopy alone, without the need for additional procedures. However, four patients required more advanced interventions. Fluoroscopy was used in conjunction with bronchoscopy in three patients, successfully aiding removal in two of them. The remaining two patients—one with a failed fluoroscopy attempt and another where it was not attempted—underwent mini-thoracotomy for needle removal. The average hospital stay for patients who had simple flexible bronchoscopic removal was 1.5 days (range 1-3 days). Those who underwent the successful combination of bronchoscopy and fluoroscopy stayed 2 and 4 days, with the latter patient being asthmatic and experiencing an exacerbation during hospitalization. The patients who required mini-thoracotomy had hospital stays of 4 and 6 days. Additionally, a single case involved a pin embedded in the lung parenchyma, which necessitated removal via mini-thoracotomy [17].

## Statistical Analysis

The meta-analysis statistic has been calculated using Jamovi software 2.4, MAJOR meta-analysis proportion section. The proportion of single arm model has been established.

## Outcomes

**Figure-4:**
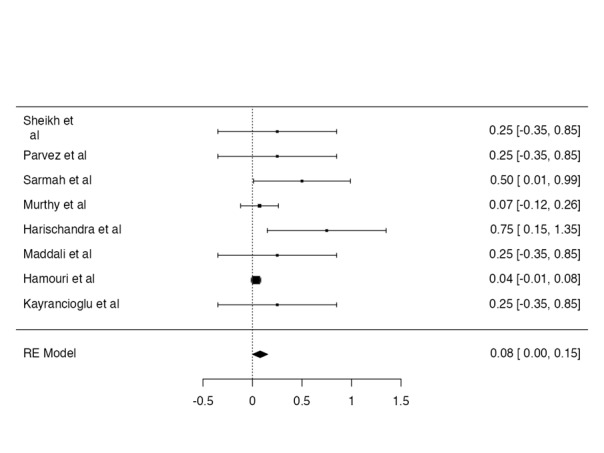
Figure 4. Meta analysis of proportion of the Thoracotomy rate (8%)

**Figure-5:**
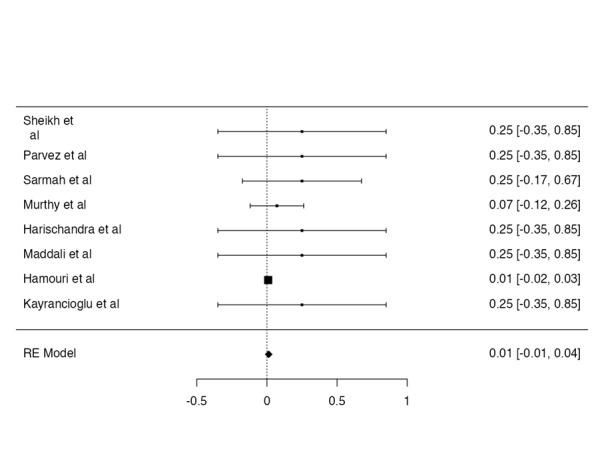
Figure 5. Meta analysis of proportion of the bleeding rate (1.4%)

Thoracotomy convertion rate

A total of 71 patients included in this review from 7 published study and 1 from our case. The thoracotomy rate was as 8%. The proportion meta-analysis forest plot was given in Figure-4.

Bleeding rate

The bleeding was reported only in 1 case. The bleeding rate was reported as 1.4% (Figure-5).

Reoperation rate

**Figure-6:**
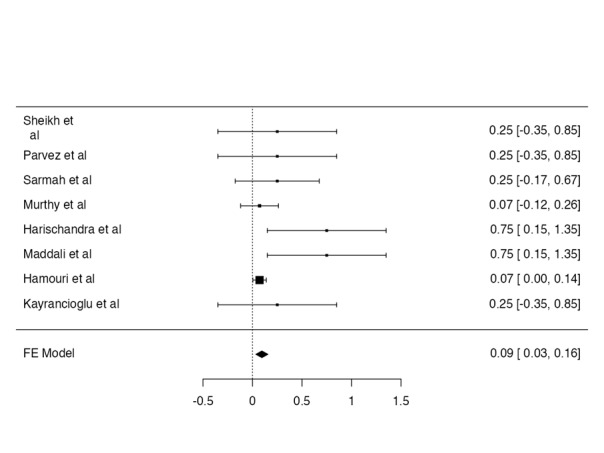
Figure 6. Meta analysis of proportion of reoperation rate rate (9.8%)

The reoperation rate was reported 7 cases. The reoperation rate was reported as 9.8% (Figure-6).

Postoperative intubation rate

**Figure-7:**
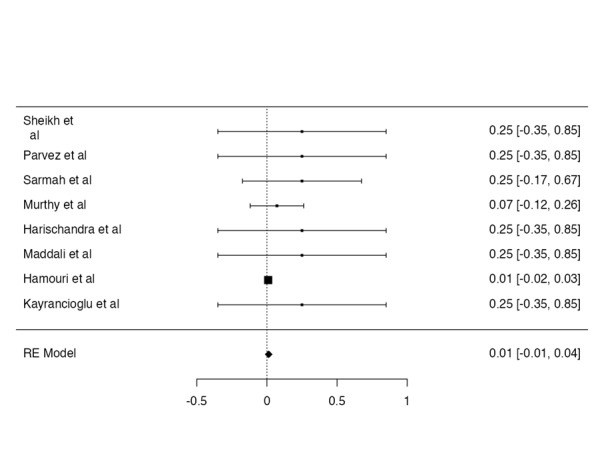
Figure 7. Meta analysis of proportion of postoperative intubation rate (1.4%)

The postoperative intubation was reported only in 1 case. The postoperative intubation rate was reported as 1.4% (Figure-7).

## Complication Analysis

**Figure-8:**
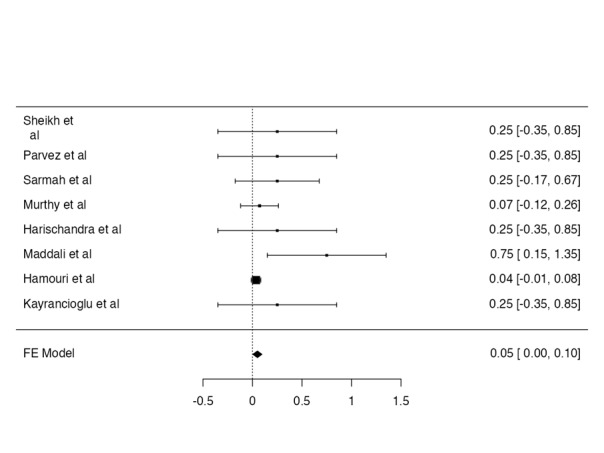
Figure 8. Meta analysis of proportion of any complication rate (5.6%)

Chest drain was inserted in 1 patients of Sarmah et al. Controlateral atelectasis was reported in 1 patient by Maddali et al. and Astma in 2 patients in the cases of Hamouri et al. The complications (any complications) were found in total of 4 patients. The calculated any complication rate was found as 5.6% based on existing literature (Figure-8).

Postoperative hemoptysis

Postoperative hemoptysis was not reported across any included studies. However, the postoperative hemoptysis incidence was calculated as 0% based on included studies.

Mortality rate

Mortality was not reported across any included studies. However, the mortality incidence was calculated as 0% based on included studies.

## Discussion

In our case, the successful removal of the pin via rigid bronchoscopy was achieved without complications. However, it is important to highlight that the shape and position of the pin presented a unique challenge. Careful maneuvering and the use of forceps without a sheath were crucial in ensuring the pin’s safe extraction.

FBA is a significant concern in pediatric populations, particularly among children under the age of two, where it remains a leading cause of morbidity and mortality. The aspiration of sharp objects, such as headscarf pins, presents unique challenges due to their potential to become lodged in the airway and cause severe complications [1][11]. In cultures where headscarf use is common, the risks associated with pin aspiration are well-documented, yet managing these cases continues to pose difficulties [12][13][14][15][16][17].

The diagnosis of foreign body aspiration is often straightforward when the patient presents with classic symptoms such as persistent cough, stridor, or wheezing [4][5]. However, some cases may present asymptomatically or with subtle signs, making clinical suspicion and prompt imaging crucial for early detection [1][6]. Chest X-rays and computed tomography (CT) scans are typically employed to identify the location and orientation of the foreign body, guiding the intervention strategy [3].

Rigid bronchoscopy remains the gold standard for the removal of foreign bodies from the airways, but it is not without challenges [1]. The sharp ends of hijab pins can become embedded in the airway mucosa, complicating their extraction. In some cases, multiple attempts with different techniques, including flexible bronchoscopy or even surgical intervention, may be necessary to safely remove the object. Even in our case we have removed the pin via RB, the meta analysis found a 8% thoractomy rate and 9.8% reoperation rate. However, thoracotomy should be considered as an option in cases where RB has failed. Thus, thoracotomy should be considered a viable option in these challenging cases to ensure the safe removal of the foreign body and prevent further complications.

Moreover, the meta-analysis reported a complication rate of 5.6%, with bleeding, reoperation, and postoperative intubation being the most common issues. Although the mortality rate was calculated at 0%, the potential for serious complications underscores the need for prompt and appropriate management. The literature highlights the importance of a multidisciplinary approach, involving pediatric surgeons, anesthetists, and pulmonologists, to address the complexities of these cases effectively.

Multidisciplinary collaboration plays a vital role in the successful management of foreign body aspiration cases. Pediatric surgeons are primarily responsible for the removal of the object, while anesthetists ensure airway control and patient stability throughout the procedure. Pulmonologists provide diagnostic insight and support postoperative respiratory care. This collaborative approach optimizes patient outcomes by addressing various clinical aspects simultaneously. However, the absence of long-term follow-up in our study is a limitation. Future research should focus on potential late-onset complications, such as recurrent respiratory infections or airway scarring, and assess long-term respiratory outcomes in children, enhancing our understanding of the broader impacts of foreign body removal procedures.

The limitations underscore the need for caution in interpreting the results of this study. The small sample size and study heterogeneity may limit the applicability of the findings across different populations and clinical settings. Additionally, the potential for publication bias and the retrospective nature of many included studies introduce uncertainties that could affect the reliability of the conclusions. The lack of long-term follow-up data is particularly concerning, as it leaves unanswered questions about the possibility of delayed complications or the long-term success of the interventions. Furthermore, the exclusion of non-English studies and those with inaccessible full texts might have led to an incomplete representation of the available evidence. Future research should focus on prospective studies with larger sample sizes and standardized protocols to provide more robust and generalizable data. Addressing these limitations will be crucial for improving the understanding and management of hijab pin aspiration in pediatric patients.

## Conclusion

In conclusion, this case report of a 19-month-old girl who aspirated a bended headscarf pin into her left bronchus underscores the complexity and potential severity of such incidents in pediatric patients. The successful retrieval of the pin using rigid bronchoscopy, without complications, highlights the effectiveness of this procedure when performed promptly and by experienced hands. However, the case also illustrates the need for careful consideration of alternative approaches, including flexible bronchoscopy and even surgical intervention, when initial attempts are unsuccessful. The systematic review and meta-analysis further reinforce the importance of early detection and intervention in preventing complications such as bleeding, reoperation, or prolonged hospital stays. Although the overall complication and mortality rates associated with pin aspiration are low, the potential for serious outcomes necessitates a high index of suspicion and a multidisciplinary approach to management. This report serves as a valuable reminder for healthcare providers to be vigilant about the risks associated with foreign body aspiration, particularly in cultures where the use of headscarf pins is prevalent, and to act swiftly to ensure optimal patient outcomes.

## Conflict of Interest

None

## Apendix 1: Search strategy

"pin"[All Fields] AND ("aspirant"[All Fields] OR "aspirants"[All Fields] OR "aspirate"[All Fields] OR "aspirates"[All Fields] OR "aspirating"[All Fields] OR "aspirational"[All Fields] OR "aspirations, psychological"[MeSH Terms] OR ("aspirations"[All Fields] AND "psychological"[All Fields]) OR "psychological aspirations"[All Fields] OR "aspirations"[All Fields] OR "aspirative"[All Fields] OR "aspirator"[All Fields] OR "aspirators"[All Fields] OR "aspire"[All Fields] OR "aspired"[All Fields] OR "aspires"[All Fields] OR "aspiring"[All Fields] OR "respiratory aspiration"[MeSH Terms] OR ("respiratory"[All Fields] AND "aspiration"[All Fields]) OR "respiratory aspiration"[All Fields] OR "aspirated"[All Fields] OR "aspiration"[All Fields] OR "aspir*"[All Fields] OR ("administration, inhalation"[MeSH Terms] OR ("administration"[All Fields] AND "inhalation"[All Fields]) OR "inhalation administration"[All Fields] OR "inhalant"[All Fields] OR "inhalability"[All Fields] OR "inhalable"[All Fields] OR "inhalants"[All Fields] OR "inhalated"[All Fields] OR "inhalation"[MeSH Terms] OR "inhalation"[All Fields] OR "inhal"[All Fields] OR "inhalations"[All Fields] OR "inhale"[All Fields] OR "inhaled"[All Fields] OR "inhaling"[All Fields] OR "inhalational"[All Fields] OR "inhalative"[All Fields] OR "inhalatively"[All Fields] OR "inhalent"[All Fields] OR "inhaler s"[All Fields] OR "inhales"[All Fields] OR "nebulizers and vaporizers"[MeSH Terms] OR ("nebulizers"[All Fields] AND "vaporizers"[All Fields]) OR "nebulizers and vaporizers"[All Fields] OR "inhalator"[All Fields] OR "inhalators"[All Fields] OR "inhaler"[All Fields] OR "inhalers"[All Fields]) OR ("administration, inhalation"[MeSH Terms] OR ("administration"[All Fields] AND "inhalation"[All Fields]) OR "inhalation administration"[All Fields] OR "inhalant"[All Fields] OR "inhalability"[All Fields] OR "inhalable"[All Fields] OR "inhalants"[All Fields] OR "inhalated"[All Fields] OR "inhalation"[MeSH Terms] OR "inhalation"[All Fields] OR "inhal"[All Fields] OR "inhalations"[All Fields] OR "inhale"[All Fields] OR "inhaled"[All Fields] OR "inhaling"[All Fields] OR "inhalational"[All Fields] OR "inhalative"[All Fields] OR "inhalatively"[All Fields] OR "inhalent"[All Fields] OR "inhaler s"[All Fields] OR "inhales"[All Fields] OR "nebulizers and vaporizers"[MeSH Terms] OR ("nebulizers"[All Fields] AND "vaporizers"[All Fields]) OR "nebulizers and vaporizers"[All Fields] OR "inhalator"[All Fields] OR "inhalators"[All Fields] OR "inhaler"[All Fields] OR "inhalers"[All Fields])) AND ("infant"[MeSH Terms] OR "infant"[All Fields] OR "infants"[All Fields] OR "infant s"[All Fields] OR ("men"[MeSH Terms] OR "men"[All Fields] OR "boy"[All Fields]) OR ("women"[MeSH Terms] OR "women"[All Fields] OR "girl"[All Fields]) OR ("infant, newborn"[MeSH Terms] OR ("infant"[All Fields] AND "newborn"[All Fields]) OR "newborn infant"[All Fields] OR "baby"[All Fields] OR "infant"[MeSH Terms] OR "infant"[All Fields]) OR ("infant, newborn"[MeSH Terms] OR ("infant"[All Fields] AND "newborn"[All Fields]) OR "newborn infant"[All Fields] OR "newborn"[All Fields] OR "newborns"[All Fields] OR "newborn s"[All Fields]) OR ("infant, newborn"[MeSH Terms] OR ("infant"[All Fields] AND "newborn"[All Fields]) OR "newborn infant"[All Fields] OR "neonatal"[All Fields] OR "neonate"[All Fields] OR "neonates"[All Fields] OR "neonatality"[All Fields] OR "neonatals"[All Fields] OR "neonate s"[All Fields]) OR ("2year"[All Fields] OR "2years"[All Fields]) OR ("twoyear"[All Fields] OR "twoyears"[All Fields]) OR ("1year"[All Fields] OR "1years"[All Fields]) OR "oneyear"[All Fields] OR ("child"[MeSH Terms] OR "child"[All Fields] OR "children"[All Fields] OR "child s"[All Fields] OR "children s"[All Fields] OR "childrens"[All Fields] OR "childs"[All Fields]))
